# Author Correction: Distinct and additive effects of calorie restriction and rapamycin in aging skeletal muscle

**DOI:** 10.1038/s41467-022-30189-8

**Published:** 2022-04-27

**Authors:** Daniel J. Ham, Anastasiya Börsch, Kathrin Chojnowska, Shuo Lin, Aurel B. Leuchtmann, Alexander S. Ham, Marco Thürkauf, Julien Delezie, Regula Furrer, Dominik Burri, Michael Sinnreich, Christoph Handschin, Lionel A. Tintignac, Mihaela Zavolan, Nitish Mittal, Markus A. Rüegg

**Affiliations:** 1 Biozentrum, University of Basel, Basel, Switzerland; 2 Department of Biomedicine, Pharmazentrum, University of Basel, Basel, Switzerland

**Keywords:** Ageing, Homeostasis, Muscle

Correction to: *Nature Communications* 10.1038/s41467-022-29714-6, published online 19 April 2022.

In this article the author name Aurel B. Leuchtmann was incorrectly written as Aurel B. Leuchtman. The original article has been corrected.

