# Supplementary material for: Distinct and additive effects of calorie restriction and rapamycin in aging skeletal muscle
Source: Nat Commun. 2022 Apr 19;13:2025. doi: 10.1038/s41467-022-29714-6 (PMC9018781; doi:10.1038/s41467-022-29714-6)
Supplement: Supplementary file 1 — Supplementary Information [file 41467_2022_29714_MOESM1_ESM.pdf]

Supplementary Information: Distinct and additive effects of calorie restriction and rapamycin in aging skeletal muscle

M. Rüegg et al.

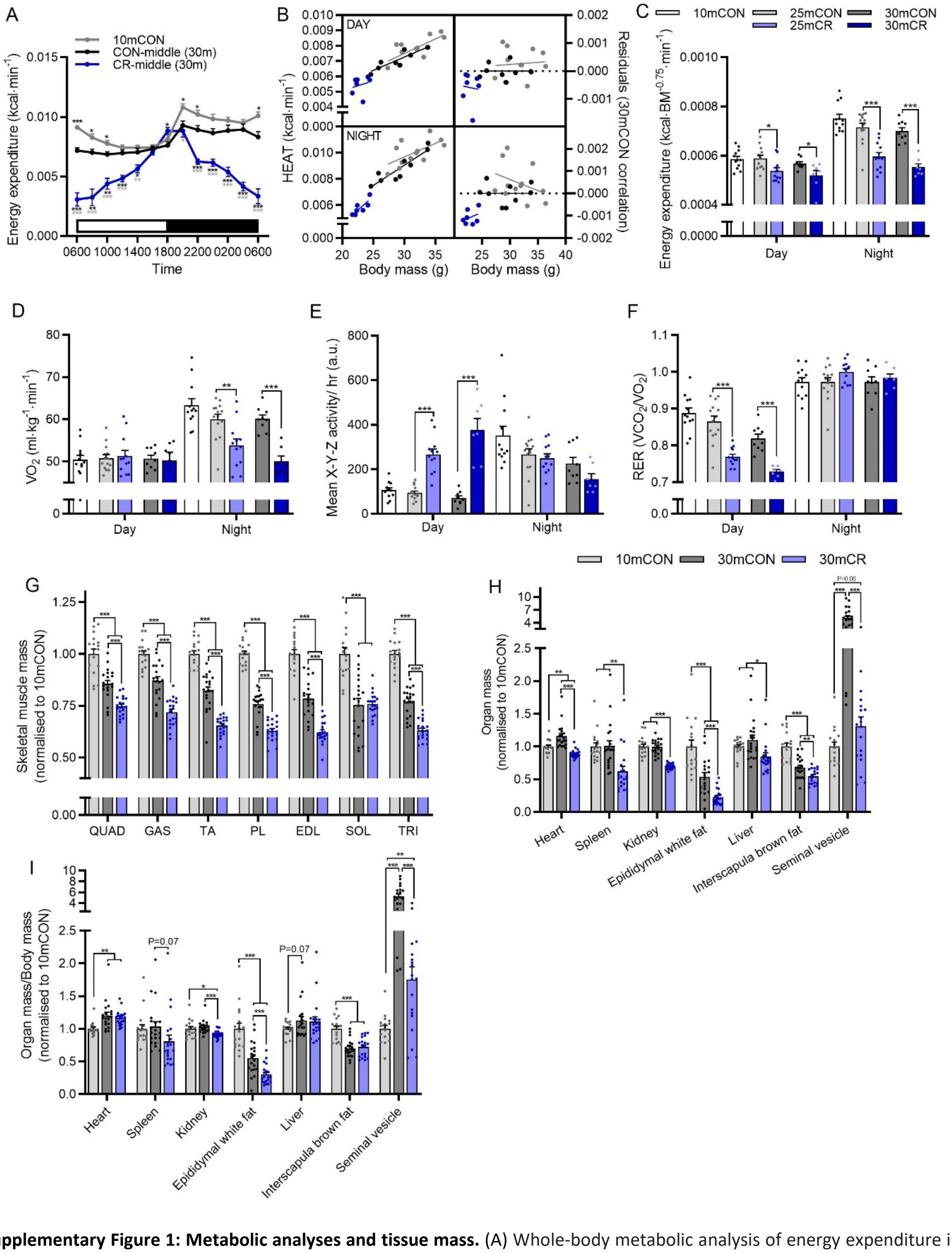

Supplementary Figure 1: Metabolic analyses and tissue mass. (A) Whole-body metabolic analysis of energy expenditure in kcal·min^{-1} reported every 2 h across one full day (white)/night (black) cycle and (B) the relationship between body mass and day or nighttime energy expenditure for 10mCON, 30mCON and 30mCR. Mean day and night whole-body metabolic analysis of (C) energy expenditure normalized to body surface area, (D) VO2 ml·kg^{-1}·min^{-1}, (E) X-Y-Z activity per hour and (F) Respiratory exchange ratio at 25 and 30-months of age for 30mCON (*n* = 14 at 25m and 9 at 30m) and 30mCR (*n* = 12 at 25m and 7 at 30m) groups as well as for 10mCON (*n* = 12) mice. (G) Skeletal muscle mass normalized to 10mCON and (H) organ mass normalized to 10m control alone or (I) to body mass and then 10mCON. Group numbers for (G-I) are 17 (10m), 20 (30mCON) and 20 (30mCR) except for brown fat and seminal vesicles (*n* = 19). Data are presented as mean ± SEM. Two-way repeated-measure ANOVA with Sidak or Tukey post hoc tests (A–F), and one-way ANOVA with Fisher's LSD post hoc tests (G-I) were used to compare the data. *, **, and *** denote a significant difference between groups of $P < 0.05$, $P < 0.01$, and $P < 0.001$, respectively. # denotes a trend where $0.05 < P < 0.10$. Colored asterisks refer to the group of comparison.

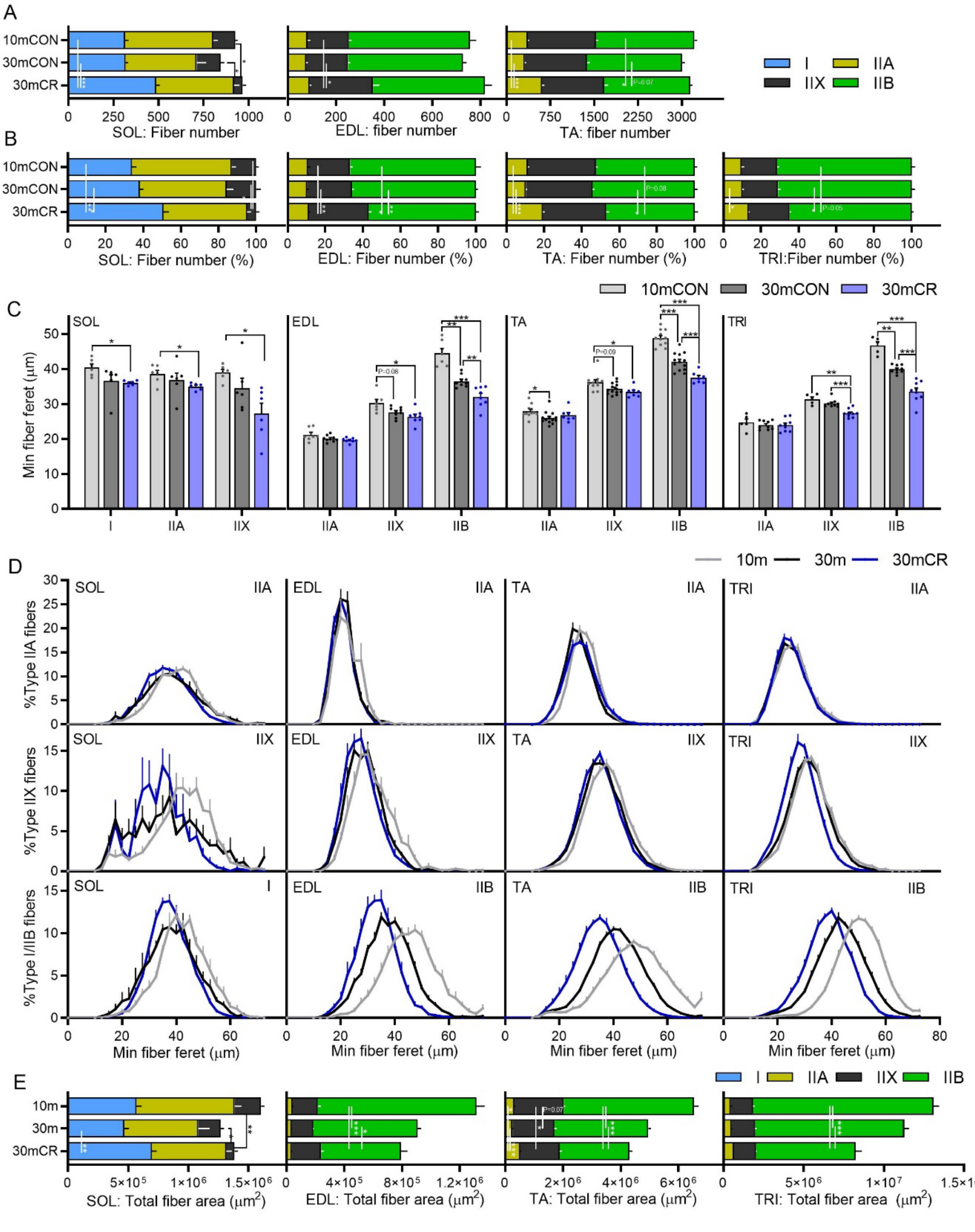

Supplementary Figure 2: CR promotes a fast-to-slow muscle fiber phenotype shift. Fiber-type-specific (A) absolute fiber counts, (B) fiber type proportions, (C) mean minimum fiber feret, fiber size distribution and (E) total fiber cross sectional area on whole cross sections from soleus (SOL; *n* = 6), extensor digitorum longus (EDL; *n* = 7, 9 and 8), tibialis anterior (TA; *n* = 11, 13 and 7) and triceps brachii (TRI; *n* = 5, 9 and 9) for 10mCON, 30mCON and 30mCR muscles stained with antibodies against type I, type IIA and type IIB fibers as well as laminin, while fibers without staining were classified as IIX. Data are presented as mean ± SEM. One-way (D) or two-way repeated-measure (A-C, E) ANOVAs with Fisher's LSD or Tukey's post hoc tests, respectively, were used to compare between data. *, **, and *** denote a significant difference between groups of $P < 0.05$, $P < 0.01$, and $P < 0.001$, respectively. Colored asterisks refer to the group of comparison.

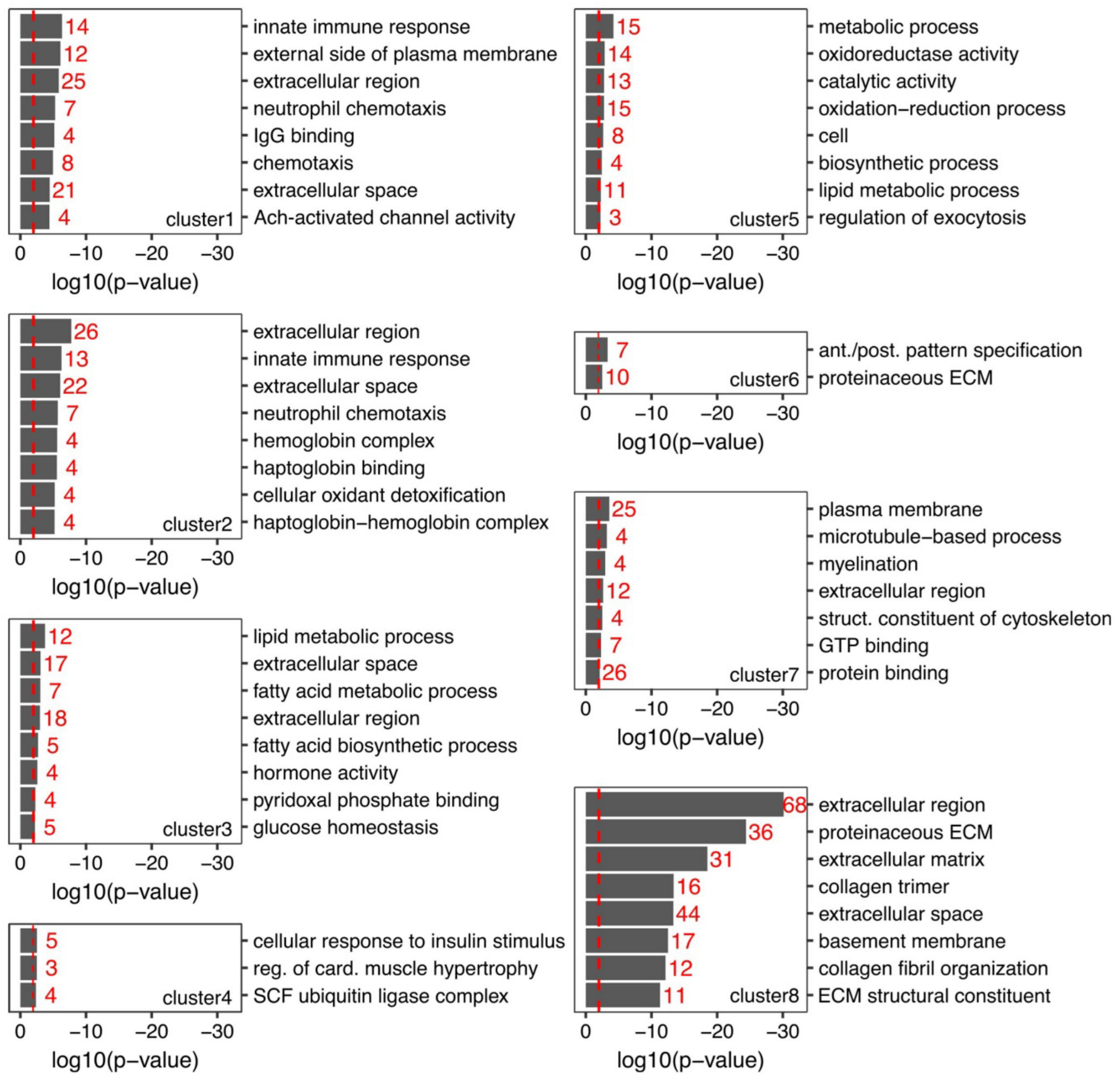

Supplementary Figure 3: Gene ontology of gene clusters. Top-eight DAVID gene ontology terms enriched ($P < 0.01$) for genes aligned to each of the eight clusters (Fig. 3E) identified through hierarchical clustering of genes aligned with any of the four PCs described in Figure 3B. Enrichment significance threshold was set at $P < 0.01$ (gray and red dashed lines). A one-sided, modified Fisher's exact test was used to determine significance.

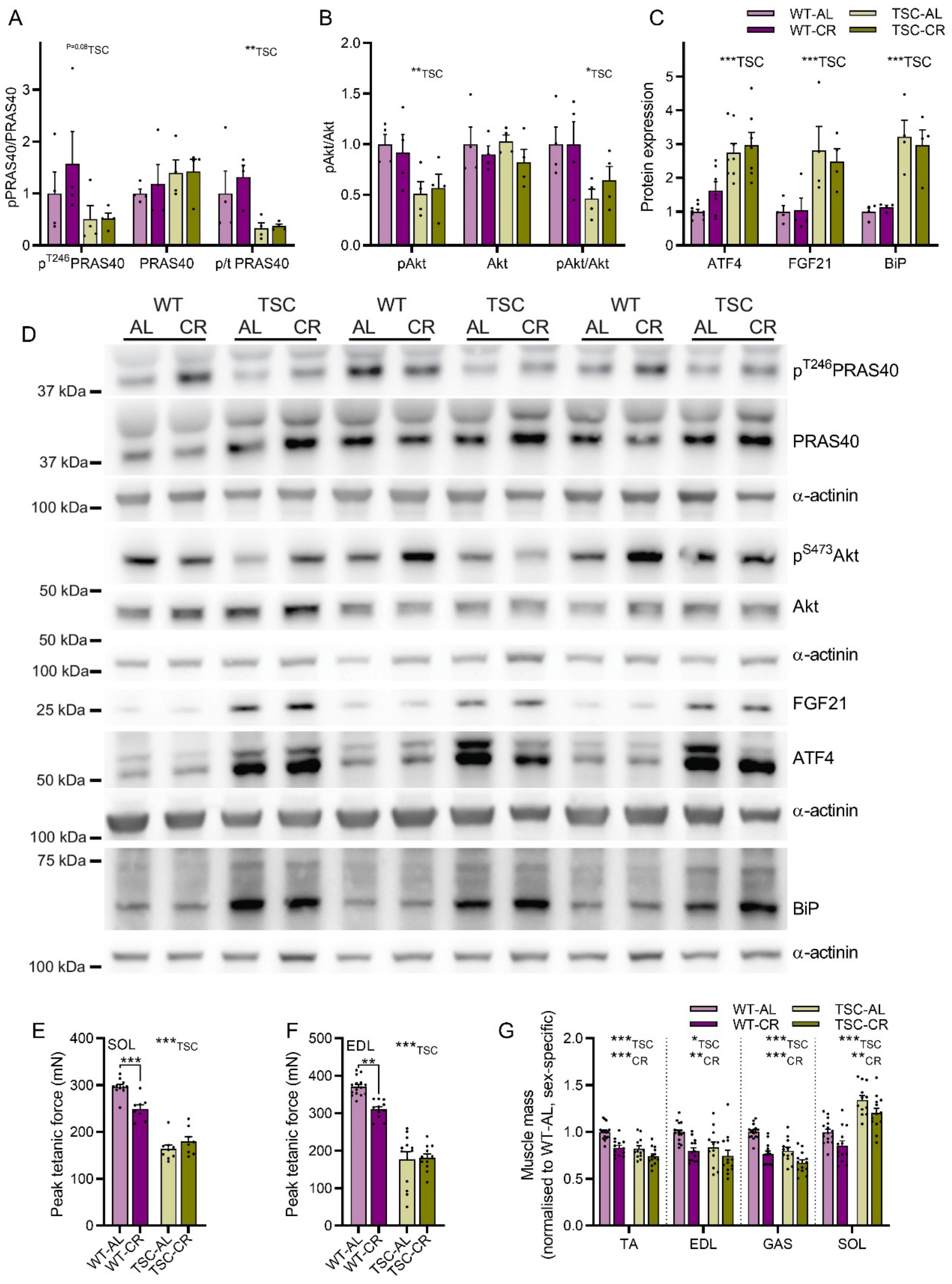

Supplementary Figure 4. CR does not alter AKT activation, ER stress induction or absolute peak tetanic force in TSCmKO mice, but reduces absolute muscle mass. Quantification of phosphorylated and total protein levels for (A) PRAS40 and (B) AKT as well as total levels of (C) ATF4, FGF21 and BiP and (D) Representative western blot images. Two independent blots were performed for ATF4 and FGF21. Absolute peak tetanic force for (E) the slow-twitch *soleus* (SOL) muscle and (F) the fast-twitch *extensor digitorum longus* (EDL) muscle. (G) Absolute muscle mass of *tibialis anterior* (TA), EDL, *gastrocnemius* (GAS) and SOL. For A-C, n = 4, 4, 4 and 4, except for ATF4 (n = 9, 7, 8, 7); for E, n = 11, 8, 9 and 8; for F, n = 15, 11, 12 and 11 and; for G, n = 16, 13, 12 and 13 for WT-AL, WT-CR, TSC-AL and TSC-CR, respectively. Data are presented as mean ± SEM. Two-way ANOVAs with Tukey post hoc tests were used to compare data. *, **, and *** denote a significant difference between groups of $P < 0.05$, $P < 0.01$, and $P < 0.001$, respectively. # denotes a trend where $0.05 < P < 0.10$. Colored asterisks refer to the group of comparison.

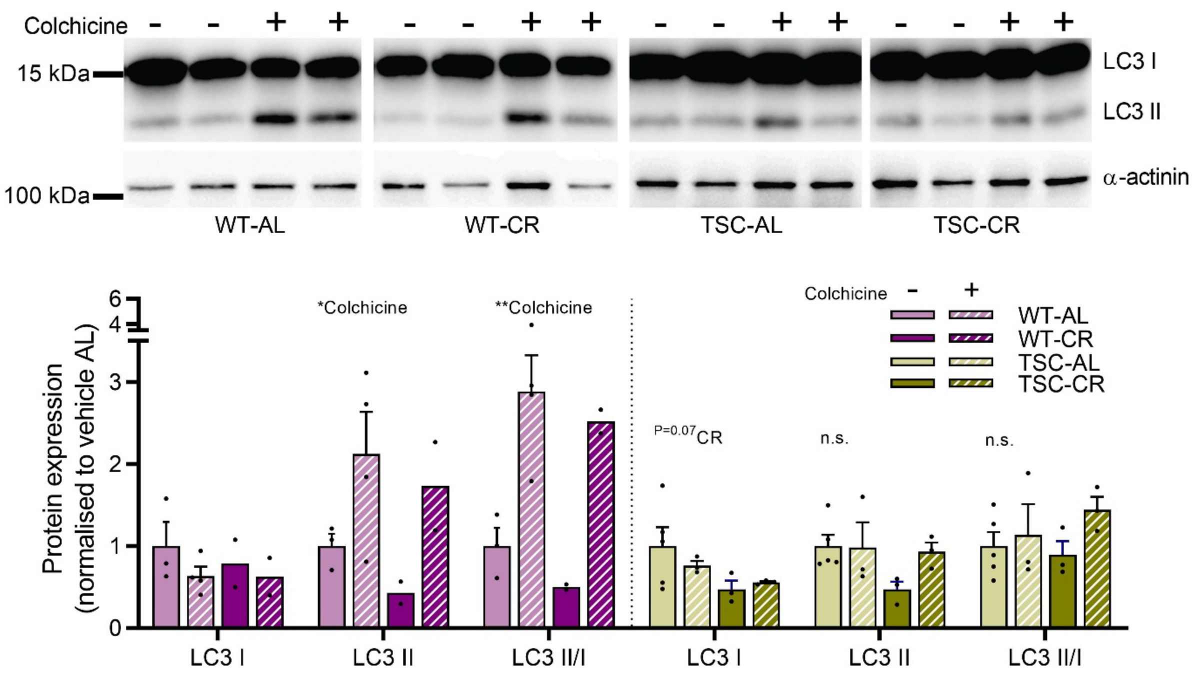

Supplementary Figure 5. CR does not restore autophagic flux in TSCmKO mice. Representative Western blots and quantification (below) of LC3 I and II in muscles from wild type (WT) and TSCmKO (TSC) mice fed ad libitum (AL) or calorie restricted (CR) for 3 months starting at 3 months of age and treated (I.P.) with either the lysosome inhibitor colchicine (0.4 mg·kg-1) or vehicle (saline) four times over a 36 h period (evening, morning, evening, morning). Tissue was collected 2-3 h after the final injection. All WT samples were ran on one blot, while all TSCmKO samples were ran on a separate blot. Group numbers are n = 3, 2, 4, 2 (vehicle) and 4, 2, 3 and 3 (Colchicine) for WT-AL, WT-CR, TSC-AL and TSC-CR, respectively. Data are presented as mean ± SEM. Two-way ANOVAs with Tukey post hoc tests were used to compare data. *, **, and *** denote a significant difference between groups of $P < 0.05$, $P < 0.01$, and $P < 0.001$, respectively. Trends are reported where $0.05 < P < 0.10$.

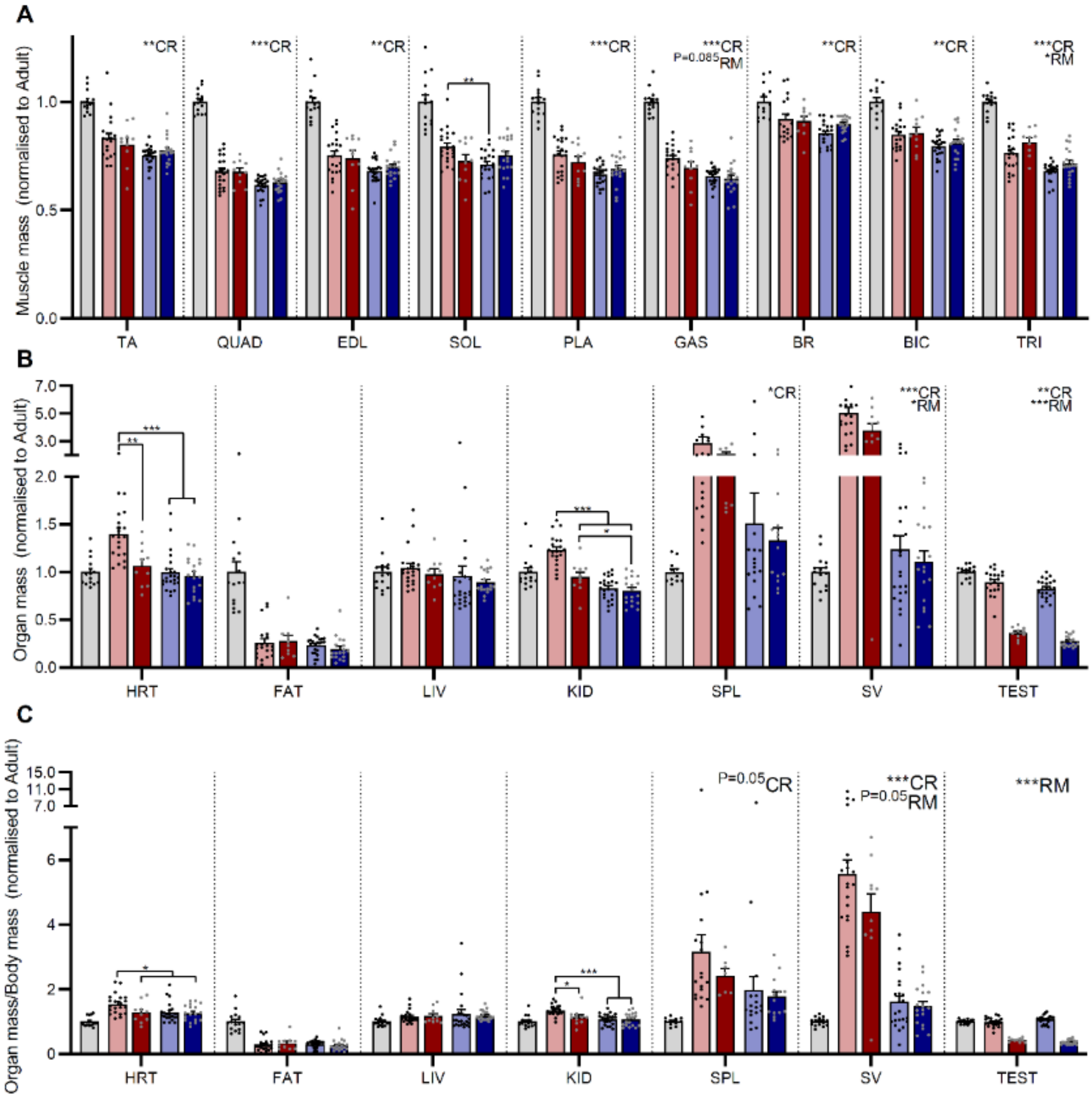

Supplementary Figure 6: CR and RM specific effects on muscle and tissue mass. (A) Muscle mass for tibialis anterior (TA), *quadriceps* (QUAD), *extensor digitorum longus* (EDL), *soleus* (SOL), *plantaris* (PLA), *gastrocnemius* (GAS), brachioradialis (BR), biceps brachii (BIC) and *triceps brachii* (TRI) were averaged across both limbs and normalized to 10-month-old control mice. Organ mass normalized to (B) 10mCON or (C) body mass and then 10mCON, including heart (HRT), epididymal fat (FAT), liver (LIV), Kidney (KID), spleen (SPL), seminal vesicles (SV) and testicles (TEST). Group numbers are n = 15 (14 for SOL, 13 for BR and BIC, 11 for spleen; Adult), 21 (17 for BR, 19 for BIC, 20 for fat and SV, 18 for spleen; CON), 10 (9 for TRI, 7 for spleen; RM), 23 (22 for SOL and BIC, 21 for BR and TRI, 17 for spleen; CR) and 19 (18 for BR, BIC, heart, fat, liver, kidney, SV and testicles, 14 for spleen; CR+RM). Data are presented as mean ± SEM. Two-way repeated- measure ANOVAs with Tukey's post hoc tests were used to compare between data. *, **, and *** denote a significant difference between groups of $P < 0.05$, $P < 0.01$, and $P < 0.001$, respectively. Colored asterisks refer to the group of comparison.

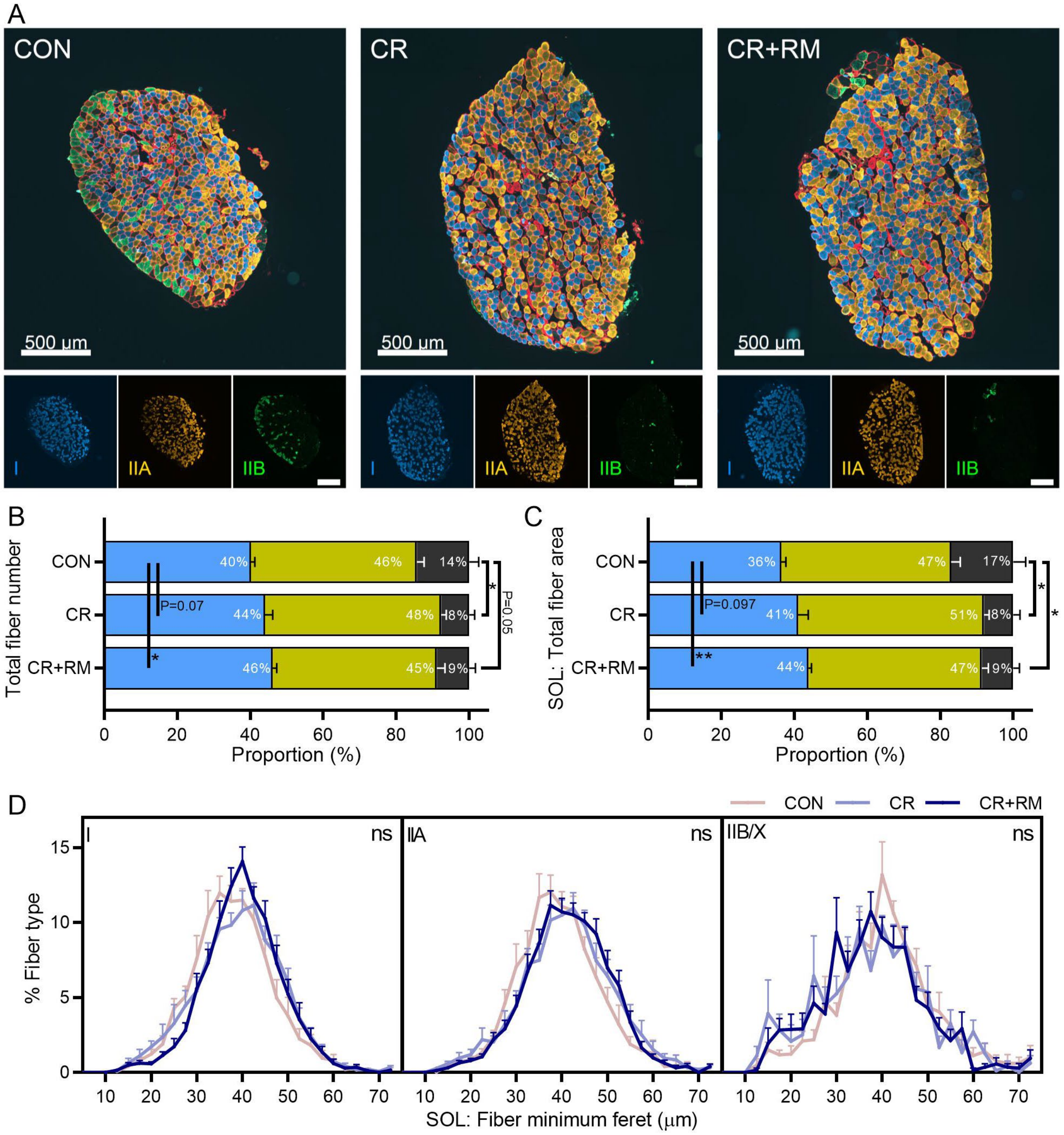

Supplementary Figure 7. CR and RM have additive effects on muscle mass, function and fiber size and composition. (A) representative images of soleus muscle cross sections from *ad libitum*-fed (CON), calorie restricted (CR) and rapamycin treated calorie restricted (CR+RM) mice stained with antibodies against type I (blue), type IIA (yellow), and type IIB (green) fibers as well as laminin (red), while fibers without staining were classified as IIX. Proportional, fiber-type specific (B) number and (C) area. (D) Fiber-type specific minimum fiber feret distribution. Data are presented as mean ± SEM; n = 8 for all groups. one-way ANOVAs with Fisher's LSD post hoc tests were used to compare between groups, so long as the ANOVA reached statistical significance. *, **, and *** denote a significant difference between groups of $P < 0.05$, $P < 0.01$, and $P < 0.001$, respectively.
