# Supplementary material for: Distinct and additive effects of calorie restriction and rapamycin in aging skeletal muscle
Source: Nat Commun. 2022 Apr 19;13:2025. doi: 10.1038/s41467-022-29714-6 (PMC9018781; doi:10.1038/s41467-022-29714-6)
Supplement: Supplementary file 2 — Reporting Summary [file 41467_2022_29714_MOESM2_ESM.pdf]

nature research

Corresponding author(s): Markus A. Rüegg, Daniel J. Ham, Nitish Mittal.

Last updated by author(s): Mar 11, 2022

Reporting Summary

Nature Research wishes to improve the reproducibility of the work that we publish. This form provides structure for consistency and transparency in reporting. For further information on Nature Research policies, see our Editorial Policies and the Editorial Policy Checklist.

Statistics

For all statistical analyses, confirm that the following items are present in the figure legend, table legend, main text, or Methods section.

n/a	Confirmed	
☐	☒	The exact sample size (n) for each experimental group/condition, given as a discrete number and unit of measurement
☒	☐	A statement on whether measurements were taken from distinct samples or whether the same sample was measured repeatedly
☐	☒	The statistical test(s) used AND whether they are one- or two-sided<br>*Only common tests should be described solely by name; describe more complex techniques in the Methods section.*
☒	☐	A description of all covariates tested
☐	☒	A description of any assumptions or corrections, such as tests of normality and adjustment for multiple comparisons
☐	☒	A full description of the statistical parameters including central tendency (e.g. means) or other basic estimates (e.g. regression coefficient) AND variation (e.g. standard deviation) or associated estimates of uncertainty (e.g. confidence intervals)
☐	☒	For null hypothesis testing, the test statistic (e.g. F, t, r) with confidence intervals, effect sizes, degrees of freedom and P value noted<br>*Give P values as exact values whenever suitable.*
☒	☐	For Bayesian analysis, information on the choice of priors and Markov chain Monte Carlo settings
☒	☐	For hierarchical and complex designs, identification of the appropriate level for tests and full reporting of outcomes
☒	☐	Estimates of effect sizes (e.g. Cohen's d, Pearson's r), indicating how they were calculated

Our web collection on statistics for biologists contains articles on many of the points above.

Software and code

Policy information about availability of computer code

Data collection	RNAseq data were processed using Cutadapt v1.9.1 and Kallisto v0.43.1. Sequences of protein coding transcripts were selected based on genome assembly GRCm38 (release 92) and transcript annotations from Ensembl database. SarcoAtlas (https://sarcoatlas.scicore.unibas.ch/) was developed previously (Ham & Börsch et al., Nat Com, 2020) using the R package Shiny v0.14.2; Code is available upon request from Dr. Anastasiya Börsch (anastasiya.boersch@unibas.ch) or any of the corresponding authors. RTqPCR Primers were designed using Geneious®10 software and specificity confirmed by the Basic Local Alignment Search Tool (BLAST). Potential hairpin formation, complementarity and self-annealing sites were verified to be negative by OligoCalc. Westerm Blot protein abundance was quantified using FusionCapt Advance (Vilber).
Data analysis	RNAseq data were analysed using the Bioconductor packages EdgeR v3.26.1 and RDAVIDWebService v1.22. Data were analysed using GraphPad Prism 8.0.2.

For manuscripts utilizing custom algorithms or software that are central to the research but not yet described in published literature, software must be made available to editors and reviewers. We strongly encourage code deposition in a community repository (e.g. GitHub). See the Nature Research guidelines for submitting code & software for further information.

Data

Policy information about availability of data

All manuscripts must include a data availability statement. This statement should provide the following information, where applicable:

- Accession codes, unique identifiers, or web links for publicly available datasets
- A list of figures that have associated raw data
- A description of any restrictions on data availability

Raw and processed RNAseq data are available at Gene Expression Omnibus (GEO) 95 under accession number GSE171322 and GSE139204. These data are also accessible using the web-based application, SarcoAtlas (https://sarcoatlas.scicore.unibas.ch/).

Field-specific reporting

Please select the one below that is the best fit for your research. If you are not sure, read the appropriate sections before making your selection.

☒ Life sciences ☐ Behavioural & social sciences ☐ Ecological, evolutionary & environmental sciences

For a reference copy of the document with all sections, see nature.com/documents/nr-reporting-summary-flat.pdf

Life sciences study design

All studies must disclose on these points even when the disclosure is negative.

Sample size	For calorie restriction studies in TSCmKO mice, sample sizes were based on previous experience in the lab working with these mice (Castets, Cell Metabolism, 2013) along with previous sample size estimation analyses (ClinCalc) indicating that 7 per group is sufficient to detect a difference between two groups of at least 15% (Power = 80%; alpha = 0.05) for measures such as in vitro muscle force that have a typical standard deviation around 10%. For long-term rapamycin studies, sample sizes took into consideration expected survival rates (~50%) along with expected variability in the extent of sarcopenia between mice.
Data exclusions	A single outlier in the 30mCON SOL group was identified in mRNA sequencing analysis and removed from further analysis based on a clear technical error.
Replication	Phenotypical responses (body mass, body composition, grip strength and voluntary running distance) to aging and calorie restriction were examined in three independent experiments starting at 15, 19 and 20 months of age. Results were reproducible between experiments. All attempts at replication were successful.
Randomization	Treatment groups were balanced based on pre-treatment measures of body mass, body composition, food intake and functional parameters.
Blinding	In vitro measurements of muscle force were blinded for genotype. Due to technical/researcher limitations and obvious phenotypes, blinding was not performed for all other experiments.

Reporting for specific materials, systems and methods

We require information from authors about some types of materials, experimental systems and methods used in many studies. Here, indicate whether each material, system or method listed is relevant to your study. If you are not sure if a list item applies to your research, read the appropriate section before selecting a response.

Materials & experimental systems

n/a	Involved in the study	
☐	☒	Antibodies
☒	☐	Eukaryotic cell lines
☒	☐	Palaeontology and archaeology
☐	☒	Animals and other organisms
☒	☐	Human research participants
☒	☐	Clinical data
☒	☐	Dual use research of concern

Methods

n/a	Involved in the study	
☒	☐	ChIP-seq
☒	☐	Flow cytometry
☒	☐	MRI-based neuroimaging

Antibodies

Antibodies used	ps2448mTOR rabbit polyclonal antibody (WB: 1:1000), Cell Signaling 2971<br>mTOR rabbit polyclonal antibody (WB: 1:1000), Cell Signaling 2972<br>p-S6S240/244 rabbit IgG monoclonal (WB: 1:1000), Cell Signaling 5364<br>p-S6S235/236 rabbit polyclonal antibody (WB: 1:1000), Cell Signaling 2211<br>S6 rabbit IgG monoclonal (WB: 1:1000), Cell Signaling 2217

pS654EBP1 (WB: 1:1000), Cell Signaling 9451
4E-BP1 rabbit polyclonal (WB: 1:1000), Cell Signaling 9452
LC3B rabbit polyclonal (WB: 1:1000), Cell Signaling 2775
pS757ULK rabbit polyclonal (WB: 1:1000), Cell Signaling 6888
ULK rabbit IgG monoclonal (WB: 1:1000), Cell Signaling 8054
Beclin1 rabbit IgG monoclonal (WB: 1:1000), Cell Signaling 3495
Bnip3 rabbit polyclonal (WB: 1:1000), Cell Signaling 3769
pT246PRAS40 rabbit IgG monoclonal (WB: 1:1000), Cell Signaling 2997
PRAS40 rabbit polyclonal (WB: 1:1000), Cell Signaling 2610
pS473AKT rabbit IgG monoclonal (WB: 1:1000), Cell Signaling 4058
Akt rabbit polyclonal (WB: 1:1000), Cell Signaling 9272
BiP rabbit IgG2b monoclonal (WB: 1:1000), Cell Signaling 3177
ATF4 rabbit IgG polyclonal (WB: 1:1000), Santa Cruz sc-200
FGF21 goat IgG polyclonal (WB: 1:500), Biotechne AF3057
α-actinin mouse IgG1 monoclonal (WB: 1:5000), Sigma A7732
p62 guinea pig polyclonal (WB: 1:1000), Progen GP62-C
Myosin 7 mouse IgG2b monoclonal (IHC: 1:50), DSHB BA-D5
Myosin 2 mouse IgG1 monoclonal (IHC: 1:200), DSHB SC-71
Myosin 4 mouse IgM monoclonal (IHC: 1:50), DSHB BF-F3
Laminin rabbit IgG polyclonal (IHC: 1:150), Abcam 11575
Laminin 2α rat IgG1 monoclonal (IHC: 1:100), Abcam 11576
Goat anti mouse polyclonal IgG2b DyLight 405 (1:50), Jackson 115-475-207
Goat anti mouse polyclonal IgG1 Alexa568 (1:100), Invitrogen A-21124
Goat anti mouse polyclonal IgM Alexa488 (1:100), Invitrogen A-21042
Donkey anti rabbit polyclonal IgG Alexa647 (1:200), Jackson 711-605-152
Donkey anti giunea pig polyclonal IgG Cy3 (1:500), Jackson 706-165-148
Goat anti rat polyclonal IgG Alexa 488 (1:500), Jackson 112-545-003

Validation

ps2448mTOR (WB: 1:1000), Cell Signaling 2971; https://www.cellsignal.com/products/primary-antibodies/phospho-mtor-ser2448-antibody/2971
mTOR (WB: 1:1000), Cell Signaling 2972; https://www.cellsignal.com//products/primary-antibodies/mtor-antibody/2972
p-S6S240/244 rabbit IgG monoclonal (WB: 1:1000), Cell Signaling 5364; https://www.cellsignal.com//products/primary-antibodies/phospho-s6-ribosomal-protein-ser240-244-d68f8-xp-rabbit-mab/5364
p-S6S235/236 (WB: 1:1000), Cell Signaling 2211; https://www.cellsignal.com//products/primary-antibodies/phospho-s6-ribosomal-protein-ser235-236-antibody/2211
S6 rabbit IgG monoclonal (WB: 1:1000), Cell Signaling 2217; https://www.cellsignal.com//products/primary-antibodies/s6-ribosomal-protein-5g10-rabbit-mab/2217
pS654EBP1 (WB: 1:1000), Cell Signaling 9451; https://www.cellsignal.com//products/primary-antibodies/phospho-4e-bp1-ser65-antibody/9451
4E-BP1 rabbit polyclonal (WB: 1:1000), Cell Signaling 9452; https://www.cellsignal.com//products/primary-antibodies/4e-bp1-antibody/9452
LC3B (WB: 1:1000), Cell Signaling 2775; https://www.cellsignal.com//products/primary-antibodies/lc3b-antibody/2775
pS757ULK (WB: 1:1000), Cell Signaling 6888; https://www.cellsignal.com//products/primary-antibodies/phospho-ulk1-ser757-antibody/6888
ULK (WB: 1:1000), Cell Signaling 8054; https://www.cellsignal.com//products/primary-antibodies/ulk1-d8h5-rabbit-mab/8054
Beclin1 (WB: 1:1000), Cell Signaling 3495; https://www.cellsignal.com//products/primary-antibodies/beclin-1-d40c5-rabbit-mab/3495
Bnip3 (WB: 1:1000), Cell Signaling 3769; https://www.cellsignal.com//products/primary-antibodies/bnip3-antibody-rodent-specific/3769
pT246PRAS40 (WB: 1:1000), Cell Signaling 2997; https://www.cellsignal.com//products/primary-antibodies/phospho-pras40-thr246-c77d7-rabbit-mab/2997
PRAS40 (WB: 1:1000), Cell Signaling 2610; https://www.cellsignal.com//products/primary-antibodies/pras40-antibody/2610
pS473AKT (WB: 1:1000), Cell Signaling 4058; https://www.cellsignal.com//products/primary-antibodies/phospho-akt-ser473-193h12-rabbit-mab/4058
Akt rabbit polyclonal (WB: 1:1000), Cell Signaling 9272; https://www.cellsignal.com//products/primary-antibodies/akt-antibody/9272
BiP (WB: 1;1000), Cell Signaling 3177; https://www.cellsignal.com//products/primary-antibodies/bip-c50b12-rabbit-mab/3177
ATF4 rabbit polyclonal (WB: 1:1000), Santa Cruz sc-200; https://www.scbt.com/p/creb-2-antibody-c-20
FGF21 (WB: 1:500), Biotechne AF3057; https://www.rndsystems.com/products/mouse-fgf-21-antibody_af3057
α-actinin mouse IgG1 EA-53 monoclonal (WB: 1:5000), Sigma A7732; https://www.sigmaaldrich.com/catalog/product/sigma/a7732
p62 guinea pig polyclonal (WB: 1:1000), Progen GP62-C; https://www.progen.com/anti-p62-sqstm1-c-terminus-guinea-pig-polyclonal-serum.html
Myosin 7 (IHC: 1:50), DSHB BA-D5; https://dshb.biology.uiowa.edu/BA-D5
Myosin 2 (IHC: 1:200), DSHB SC-71; https://dshb.biology.uiowa.edu/SC-71
Myosin 4 (IHC: 1:50), DSHB BF-F3; https://dshb.biology.uiowa.edu/BF-F3
Laminin (IHC: 1:150), Abcam 11575 ; https://www.abcam.com/laminin-antibody-ab11575.html
Laminin 2α (IHC: 1:100), Abcam 11576 ; https://www.abcam.com/laminin-2-alpha-antibody-4h8-2-ab11576.html
Goat anti mouse polyclonal IgG2b DyLight 405 (1:50), Jackson 115-475-207 https://www.jacksonimmuno.com/catalog/products/115-475-207
Goat anti mouse polyclonal IgG1 Alexa568 (1:100), Invitrogen A-21124 https://www.thermofisher.com/antibody/product/Goat-anti-Mouse-IgG1-Cross-Adsorbed-Secondary-Antibody-Polyclonal/A-21124

Goat anti mouse polyclonal IgM Alexa488 (1:100), Invitrogen A-21042 https://www.thermofisher.com/antibody/product/Goat-anti-Mouse-IgM-Heavy-chain-Cross-Adsorbed-Secondary-Antibody-Polyclonal/A-21042
Donkey anti rabbit polyclonal IgG Alexa647 (1:200), Jackson 711-605-152 https://www.jacksonimmuno.com/catalog/products/711-605-152
Donkey anti giunea pig polyclonal IgG Cy3 (1:500), Jackson 706-165-148 https://www.jacksonimmuno.com/catalog/products/706-165-148
Goat anti rat polyclonal IgG Alexa 488 (1:500), Jackson 112-545-003 https://www.jacksonimmuno.com/catalog/products/112-545-003

Animals and other organisms

Policy information about studies involving animals; ARRIVE guidelines recommended for reporting animal research

Laboratory animals	C57BL/6JRj male mice were purchased from the aging colony at Janvier Labs. TSCmKO mice (male and female) and WT controls were bred on a C57BL/6JRj background. Wild-type mice used for long term CR studies were examined between 10 and 31 months of age. TSCmKO mice were 11 months old. Mice were kept on a 12 hr light-dark cycle (6 am to 6 pm) at 22°C (range 20-24°C) and 55% (range 45-65%) relative humidity.
Wild animals	This study did not involve wild animals
Field-collected samples	This study did not involve field-collected samples
Ethics oversight	All experiments were approved by the regional animal ethics Committee of Basel-Stadt, Switzerland.

Note that full information on the approval of the study protocol must also be provided in the manuscript.
